# Mechanisms of Neuroendocrine Stress Response in *Drosophila* and Its Effect on Carbohydrate and Lipid Metabolism

**DOI:** 10.3390/insects14050474

**Published:** 2023-05-17

**Authors:** Margarita A. Bobrovskikh, Nataly E. Gruntenko

**Affiliations:** Institute of Cytology and Genetics SB RAS, 630090 Novosibirsk, Russia; eremina@bionet.nsc.ru

**Keywords:** *Drosophila melanogaster*, insulin signaling pathway, juvenile hormone, 20-hydroxyecdysone, biogenic amines, adipokinetic hormone, stress, trehalose and glucose, lipid stores, feeding behavior

## Abstract

**Simple Summary:**

The response of living beings to adverse conditions, known as the stress reaction, is a complex mechanism including various signaling pathways and hormones. Some are evolutionarily conserved, such as the insulin signaling pathway, others, such as 20-hydroxyecdysone, adipokinetic or juvenile hormones, are taxon-specific in insects. Here we try to elucidate their interaction on the *Drosophila melanogaster* model and put together all data on the subject that currently exist in the scientific literature, creating a detailed, coherent picture. We also discuss possible mechanisms which allow stress-related hormones and insulin-like peptides to regulate feeding behavior and carbohydrate and lipid metabolism in *D. melanogaster* imagoes under heat stress conditions.

**Abstract:**

Response to short-term stress is a fundamental survival mechanism ensuring protection and adaptation in adverse environments. Key components of the neuroendocrine stress reaction in insects are stress-related hormones, including biogenic amines (dopamine and octopamine), juvenile hormone, 20-hydroxyecdysone, adipokinetic hormone and insulin-like peptides. In this review we focus on different aspects of the mechanism of the neuroendocrine stress reaction in insects on the *D. melanogaster* model, discuss the interaction of components of the insulin/insulin-like growth factors signaling pathway and other stress-related hormones, and suggest a detailed scheme of their possible interaction and effect on carbohydrate and lipid metabolism under short-term heat stress. The effect of short-term heat stress on metabolic behavior and possible regulation of its mechanisms are also discussed here.

## 1. Introduction 

Adverse environmental effects on living beings launch a series of reactions on the cellular, neuroendocrine and behavioral levels, which leads to the activation of defense processes and enhances adaptation. In insects, the neuroendocrine stress reaction is currently considered to include the following elements: the insulin signaling pathway, biogenic amines, dopamine and octopamine, functioning as both neuromediators and neurohormones, the neuropeptide adipokinetic hormone, as well as 20-hydroxyecdysone and the juvenile hormone–two hormones controlling larvae development, metamorphosis and reproduction. Disruption of any of the components of the neuroendocrine stress reaction can influence insect stress resistance. Here, we attempted to present a coherent view of the interplay of these components under stress of varying duration on the *Drosophila melanogaster* model. 

## 2. Insulin/Insulin-Like Growth Factors Signaling Pathway in *Drosophila melanogaster*

The insulin/insulin-like growth factors signaling (IIS) pathway is evolutionarily conserved among all metazoans and performs a vital role in the regulation of growth, development, reproduction, longevity, metabolism and stress resistance [1,2]. In *D. melanogaster*, eight insulin-like peptides (DILP1-8) have been identified: DILP1-5 show significant homology with insulin, DILP6–with insulin-like growth factors, DILP7 and DILP8–with mammalian relaxins [3,4,5,6,7]. 

DILPs are produced in medial neurosecretory cells or insulin-producing cells (IPCs) of the brain, as well as in the cells of peripheral tissues such as the visceral muscles of the gut, the fat body, which is the main metabolic organ in insects, neurons of the abdominal ganglia, and ovaries in a tissue- and stage-specific way [3,6,8,9,10,11,12]. Neuronal DILPs are secreted into the hemolymph and received by a homolog of the insulin receptor (dInR) for transmitting its signals to target cells [8,13,14,15,16]. DILP7 acts on the Lgr4 receptor bound to G-protein and containing leucine-rich repeats, and DILP8 binds Lgr3 [17,18].

dInR is localized in numerous fly tissues, including the fat body, the endocrine gland *corpus allatum* (*CA*) and follicular cells of the ovaries [19,20,21]. Activation of dInR, directly or through an orthologue of the mammalian insulin receptor substrate (CHICO), launches the kinase cascade, and dAkt/PKB (proteinkinase B homolog) inhibits the transfer of the transcription factor of the *Drosophila* Forkhead box class O family (dFOXO) into the cell nucleus and provokes its return from the nucleus back to the cytoplasm [22]. The main localization of dFOXO in *D. melanogaster* is the fat body of the head and abdomen [23]. dFOXO plays the role of the main regulator of expression of the downstream genes participating in the metabolism, the cell cycle, the stress response, the control of longevity and apoptosis [24,25,26,27]. It has been shown that a mutant dFOXO lacking dAkt phosphorylation sites does not react to IIS inhibition, remains in the nucleus and is constitutively active [28].

## 3. Stress-Related Hormones in *Drosophila melanogaster*

In *D. melanogaster*, IPCs are similar to vertebrate pancreatic β-cells secreting insulin in response to hyperglycemia, and the role of pancreatic α-cells secreting glucagon in response to hypoglycemia is performed by the cells of the *corpus cardiacum* (*CC*) gland, which produce a glucagon-like neuropeptide, the adipokinetic hormone (AKH) [3,29,30,31]. Due to the similarity of their functions to those of α- and β-cells, IPCs and *CC*, taken together, are seen as the *Drosophila* analogue of the mammalian pancreatic gland [32].

AKH regulates metabolic response to stress, stimulating catabolic reactions and mobilizing energy stores, especially lipids and trehalose, the latter being the main carbohydrate in insects [33,34]. It has been discovered that AKH deficit leads to obesity and a decrease in the carbohydrate level in *D. melanogaster* imagoes [35,36], and flies with an *Akh* mutation have much lower carbohydrate levels in hemolymph, including trehalose, and are resistant to starvation [34]. It has also been shown that *Akh* expression and AKH content in the cells are under DILP2 regulation in *D. melanogaster* females [11].

The IIS pathway interacts with other key hormones of the insect neuroendocrine stress reaction: 20-hydroxyecdysone (20E) and juvenile hormone (JH), which play a decisive role in growth, development, molting and metamorphosis in larvae, and perform the function of gonadotropins in imagoes, as well as biogenic amines dopamine (DA) and octopamine (OA) [22].

The central location of the OA and DA synthesis is octopamine- and dopaminergic neurons of the brain, the location of the JH synthesis is the *CA* gland [37,38]. It has been established that ecdysteroid biosynthesis during development takes place in the prothoracic gland, and the ovaries serve as the main source of ecdysteroids in imagoes [21,37,39]; seminal glands also contain ecdysteroids, but there is no sufficient evidence of an entire *de novo* biosynthesis pathway in this tissue [39]. In insects, DA and OA play the role of neurotransmitters in the synaptic cleft, neuromodulators within the bounds of one tissue, and neurohormones when being transmitted by the flow of the hemolymph to large distances [40]. It has been shown that they control the activity of the endocrine glands, arousal, desensitization of sensory inputs, various complex behavior forms such as memory and learning, and mobilization of lipids and carbohydrates [40,41,42].

DA is known to participate in JH level regulation, increasing it in young females, and decreasing it in mature ones [43]. Moreover, its regulation has a feedback loop: JH lowers the DA level in young females and increases it in mature ones. DA also regulates the 20E level, increasing it in young females and decreasing it in mature ones; however, no negative relationship has been discovered–20E increases the DA level in young females and decreases it in mature ones. In turn, DA and OA influence the 20E level indirectly through the JH metabolic system. This influence is unidirectional in young females, where it increases the 20E level, and multidirectional in mature ones: OA increases the 20E level, and DA decreases it [43]. Under unfavorable conditions of varying nature, the levels of all these hormones in *Drosophila* imagoes increase sharply (accompanied by a decrease in the activity of their metabolic enzymes; Table S1), affecting survival, fecundity and longevity [22]. In larvae, stress reaction develops as an inhibition of prothoracicotropic hormone (PTTH) secretion, which leads to a delay in ecdysone secretion and an increase in JH content, resulting in delayed metamorphosis or additional molting and allowing to “wait out” unfavorable conditions [44]. OA and DA levels in larval insects have also been shown to increase under heat stress [44,45]. 

## 4. *Drosophila* IIS Pathway Regulation under Stress

In *D. melanogaster*, dFOXO transfer to the nucleus is found under oxidative and metabolic stress [46,47]; moreover, there are data implying that under heat stress dFOXO translocation to the nucleus is accompanied by an increase in its gene’s expression level in imagoes [26,48]. Sixty minutes later dFOXO translocates from the nucleus back to the cytoplasm [26].

It has been shown that dFOXO positively regulates *dilp6* mRNA level in the fat body of *Drosophila* imagoes [9]. So, *dilp6* expression is not induced in dFOXO-mutant larvae under starvation [49], and dFOXO’s effect on the expression of DILPs produced in IPCs is blocked by simultaneous repression of *dilp6* by RNA interference in the fat body [9]. At the same time, an increase in *dilp6* expression in flies with mutations in *dilp2-3*,*5* has been demonstrated [4]. Thus, DILP6 seems to connect dFOXO, the fat tissue and the endocrine function of the brain [4,9]. However, it has been shown that although the increase in *dilp6* expression observed during pupariation is delayed in larvae with a null mutation of *dfoxo*, the level of *dilp6* transcripts ultimately reaches intermediate levels; i.e., although dFOXO is necessary for timely *dilp6* expression during development, its activation can also be caused by other factors [50]. Such a factor could be 20E, as it has been shown to induce *dilp6* expression directly in the fat body at the last instar larvae [51].

However, under heat stress, dFOXO leads not to the activation, but to the inhibition of *dilp6*, which, in turn, results in *dInR* expression activation in imagoes [48]. This apparent contradiction can be explained by the existence of a feedback loop including other components of the insect neuroendocrine stress reaction, which ensure the increase in *dilp6* expression level in response to *dfoxo* mutations or prolonged exposure to such a factor as starvation. The cardinal difference between IIS pathway regulation under starvation in larvae and under heat stress in imagoes is also demonstrated by the fact that there is a decrease in *dilps* expression level in larval IPCs under starvation [50], whereas under heat stress there is an increase in the adult DILP3 production [21].

In imagoes, *dilp6* has been shown to inhibit the expression of *dilps* in IPCs and DILP2 secretion into the hemolymph [9], and *dilp6*^41^ mutants have been found to demonstrate a sharp increase in the intensity of DILP3 production under normal conditions [21], which also agrees with our hypothesis of signal transduction from dFOXO to DILP3 through DILP6 under heat stress. The fact that DILP3 then activates dInR, thus preparing the cell to respond to stress conditions, is supported by our data on the lack of change in *dInR* expression level under heat stress in flies with *dilp6* and *dfoxo* mutations, in contrast with the *w*^1118^ control line, which demonstrates an increase in *dInR* expression level in response to heat stress [48].

It is notable that not all genes of the IIS pathway participate in the response to short-term heat stress, which implies the complexity of the mechanism of insulin regulation of an organism’s response to adverse conditions. Thus, although it has been shown that in *D. melanogaster* imagoes *dilp6* transcription correlates with a decrease in DILP2 secretion, which leads to reduced insulin signaling under starvation [49], under heat stress the amount of DILP2 in IPCs does not change (unlike DILP3) [21], which indicates that DILP2 is not involved in the response to heat stress in imagoes.

**Figure 1:**
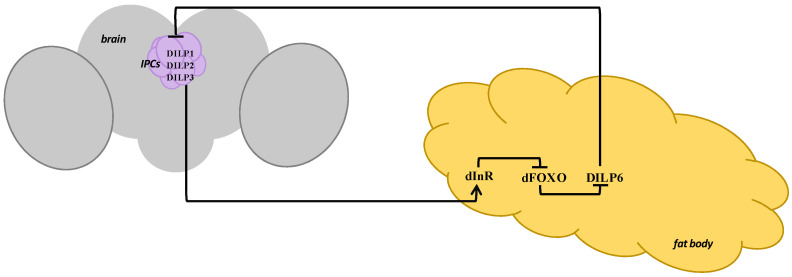
The scheme of interaction of the key components of the IIS pathway in *Drosophila* females under short-term heat stress (the “lesser” feedback loop). IPCs—insulin-producing cells, DILP—*Drosophila melanogaster* insulin-like peptide, dInR—*Drosophila melanogaster* insulin-like receptor, dFOXO—*Drosophila melanogaster* Forkhead box O transcription factor.

To sum up, a “lesser” feedback loop to DILP6 suppression by dFOXO, which has been activated under heat stress, is formed: decreased DILP6 stops inhibiting the synthesis of DILPs in IPCs, they activate the IIS in the fat body through dInR, which results in dFOXO transduction from the nucleus back to the cytoplasm (Figure 1). At the same time, it can be assumed that there is also a “greater” feedback loop activating under prolonged stress exposure—DILP6 regulation via other stress-related hormones. 

## 5. Interaction of the IIS Pathway and Stress-Related Hormones in *Drosophila melanogaster*

It has been discovered that 20E negatively affects general insulin signaling in *D. melanogaster* larvae, facilitating dFOXO transfer to the nucleus in the cells of the fat body through the activation of the hormone receptor (EcR) [52]. These results agree with the data on the increase in dFOXO transcriptional activity through 20E in the silkworm, *Bombyx mori* [53]. It has also been demonstrated that *dilp6* expression increases under the influence of 20E even in the absence of dFOXO in third instar larva [50]; it can be assumed that dFOXO activation under the influence of 20E is mediated through IIS suppression by DILP6.

In imagoes, 20E, in turn, is regulated by JH [43], which is synthesized in *CA* under the regulation of DILPs via the activation of dInR [19]. dInR localization in follicular cells of the ovaries also suggests the existence of direct regulation of the 20E synthesis by the IIS pathway [21]. The JH level in *D. melanogaster* imagoes is also regulated by its degradation enzymes, the most important of which, the JH epoxide hydrolase, is synthesized in the fat body, ovaries and gut [54,55]; it is also, apparently, controlled by dFOXO: a decrease in the enzyme’s activity has been shown both under starvation [56] and in the case of a *dfoxo* mutation [57]. The influence of this effect on DA metabolism can be negated by JH treatment of the flies [26], which is in agreement with the existence of a feedback loop in DA regulation of the JH level [43]. There is also a feedback loop in the interaction of JH and dFOXO: JH suppresses dFOXO as the activity of the latter in *D. melanogaster* larvae with an ablated *CA* increases in comparison to the control [58]. It is possible that this feedback loop is mediated by 20E activation of dFOXO in the fat body [52].

**Figure 2:**
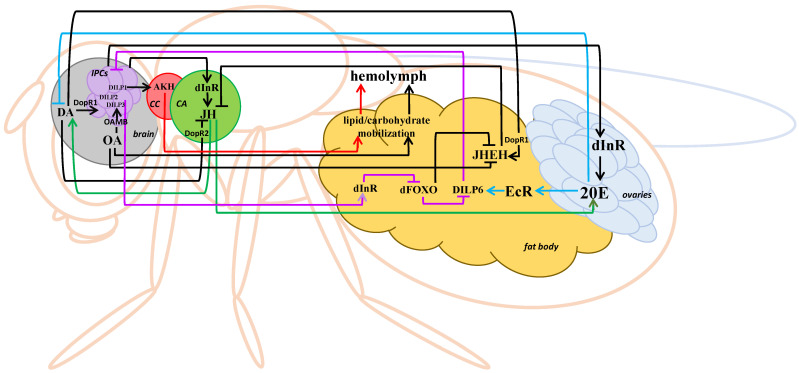
The scheme of interaction of the key components of the IIS pathway and stress-related hormones in mature *Drosophila* females under short-term heat stress. Blue arrows—the “lesser” feedback loop, black arrows—the “greater” feedback loop. IPCs—insulin-producing cells, DILP—*Drosophila melanogaster* insulin-like peptide, dInR—*Drosophila melanogaster* insulin-like receptor, dFOXO—*Drosophila melanogaster* Forkhead box O transcription factor, *CC—corpus cardiacum*, *CA—corpus allatum*, AKH—adipokinetic hormone, JH—juvenile hormone, JHEH—JH epoxide hydrolase, 20E—20-hydroxyecdysone, EcR—ecdysone receptor, DA—dopamine, DopR1—dopamine receptor mediating excitatory neurotransmission, DopR2—dopamine receptor mediating inhibitory neurotransmission, OA—octopamine, OAMB—octopamine receptor.

Notably, in imagoes, a decrease in the activity of JH epoxide hydrolase and JH esterase under nutritional stress occurs at least after 6 h of starvation and lasts at least 24 h [56], which, apparently, results in an increase in the 20E level [59] under the influence of the increased JH level and a subsequent positive regulation of DILP6. For this reason, after overnight starvation, similar to the experiments described in [50], the DILP6 level increases, which is the last stage of the “greater” feedback loop of DILP6 expression regulation by dFOXO (Figure 2). 

Another two elements of this “greater” loop, DA and OA, the levels of which also increase under stress [22], participate in the activation of DILPs production in IPCs and the regulation of the JH level. In imagoes, OA stimulates IPCs activity by binding with the OAMB receptor [60,61] and suppresses the activity of JH degradation enzymes [62,63]; at the same time, DA activates IPCs through Dopamine Receptor 1 (DopR1) [64] and decreases the JH level via the activation of the corresponding receptors in *CA* (DopR2) and the fat body (DopR1) in mature females [65]. The opposite action of the two amines on the JH metabolism may normalize the hormone’s level after the stress is over. 

It is worth noting that DILPs affect the metabolism of stress-related hormones as well. So, it has been demonstrated that a *dInR* mutation or a *dInR* knockdown in *CA* decreases the 20E and JH synthesis in *D. melanogaster* imagoes [19,66,67,68] and increases JH degradation [69]. This agrees with the data on insulin injection causing a decrease in JH degradation and an increase in the activity of the first DA synthesis enzyme, the tyrosine hydroxylase, in *D. melanogaster* females [70].

## 6. A Possible Mechanism of Short-Term Heat Stress Influence on Carbohydrate and Lipid Metabolism in *Drosophila melanogaster*

It is known that systemic defects in the IIS pathway cause a complex set of phenotypes in *D. melanogaster* including those connected to metabolism, which usually include an increase in carbohydrate and lipid stores [71]. It has been shown that most viable mutant combinations with a partial loss of function or hypomorphism of the IIS pathway genes have changes in the carbohydrate and lipid level [72]. *D. melanogaster* imagoes with *dilp6* or *dfoxo* mutations are characterized by elevated levels of glucose and trehalose [73], as well as total lipids [48], and larvae with a *dilp6* knockdown have increased levels of triglycerides and glycogen [50]. 

It has been shown that short-term heat stress causes an increase in both trehalose and glucose content in *D. melanogaster* females after just 30 min of exposure [73,74]. This agrees with earlier data on the ability of trehalose to increase resistance to temperature stress, demonstrated in the larvae of the Antarctic midge, *Belgica antarctica* [75]. Total lipid content in *D. melanogaster* imagoes also changes after heat exposure, although it occurs only 24 h later [48,76].

An immediate effect of short-term heat stress on carbohydrate but not lipid content in *D. melanogaster* females can be explained by a high solubility of carbohydrates, meaning they can be used for maintaining vital functions in rapidly changing environmental conditions, unlike lipids used by the organism as the last energy reserve under prolonged starvation or other lasting adverse influences [76]. Trehalose is known to be the main fuel for insect flight and the source of energy during nonfeeding periods [77] and thus its increase under stress provides an insect with additional energy, which allows it to avoid and/or survive adverse conditions. The assumption regarding the mobilization of lipid stores under prolonged stress is confirmed by the data on a decrease in total lipid content in *D. melanogaster* females in 24 h after heat exposure (38 °C for 60 min) [48] as well as a decrease in triglycerides content in *D. melanogaster* males in 24 h after short-term heat stress (38 °C for 45 min); curiously, this effect lasts for up to 5 days [76]. Moreover, a similar decrease in triglycerides content has been observed in *D. melanogaster* males in 24 h after short-term cold stress (4 °C, 4 h or 0 °C, 4 h) [76], which signifies a certain universality of this response.

Regarding the possible mechanisms behind the carbohydrate and lipid changes following acute heat stress, it could be assumed that the quick increase in trehalose and glucose levels is a result of increased synthesis, whereas the decrease in total lipid content results from the decrease in food consumption, which also occurs in imagoes only 24 h after the stress exposure [48]. 

It is worth noting that although *dilp6* and *dfoxo* mutations disturb the total lipid response to heat stress in imagoes, they do not prevent an increase in the carbohydrate level under stress [48,73], which signifies that the IIS pathway is not the only mechanism of the carbohydrate metabolism regulation under stress. OA likely plays the role of an additional regulator in insects when it is released into the hemolymph as a neurohormone and further transported to a target tissue, where it mobilizes lipids and carbohydrates [78,79]; other stress-related hormones, such as JH, 20E and DA, also play this part [74,76]. The largest amount of data on this have been obtained for 20E on different insect species: it stimulates glycolysis increasing the glucose level in hemolymph in the honeybee, *Apis mellifera*, larvae [80], as well as in pupae of the cotton bollworm, *Helicoverpa armigera,* [81] and the Chinese oak silk moth, *Antheraea pernyi*, [82]; it also increases lipolysis and inhibits lipid synthesis in the fall webworm, *Hyphantria cunea*, larvae [83]. 

**Figure 3:**
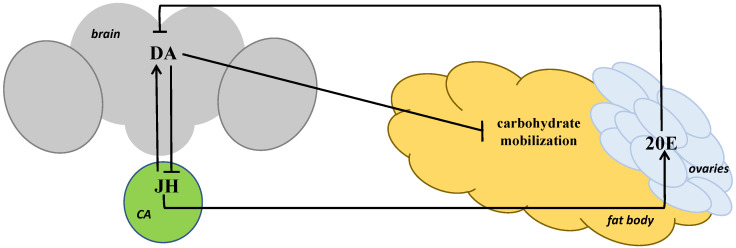
The scheme of interaction of the key stress-related hormones in *Drosophila* females under short-term heat stress. *CA—corpus allatum*, JH—juvenile hormone, 20E—20-hydroxyecdysone, DA—dopamine.

So, an increase in the DA or JH level in *D. melanogaster* females results in a decrease in trehalose and glucose content [74]; it can be assumed that they take part in normalizing the carbohydrates level after the stress is over (Figure 3). At the same time, 20E has an opposite effect on the content of the two carbohydrates [84]; it can be assumed that 20E effects the carbohydrate metabolism indirectly via DA by lowering its level, as has been shown before [43], and thus increasing the carbohydrate level, because a DA increase leads to the opposite effect on the level of both carbohydrates compared to 20E—their decrease [74]. We also assume DA to mediate the influence on the carbohydrate metabolism of not only 20E but also JH, which promotes an increase in the amine level in mature *Drosophila* females [46]. The data regarding the increase in trehalose content in *Drosophila* imagoes caused by JH application to flies [74] agree with the data received on imagoes of the red flour beetle, *Tribolium castaneum*: a decrease in the expression of genes coding a key enzyme in the JH synthesis, JH acid methyltransferase (JHAMT), and its receptor, methoprene-tolerant (Met), led to an increase in the trehalose content in hemolymph under starvation [85].

To sum up, the data on the DA, JH and 20E effect on the carbohydrate content in *D. melanogaster* [74,84] and the data on the key role of AKH in triglycerides catabolism and trehalose mobilization from glycogen [11,33,34,35,36] provide evidence that DA, the level of which is regulated positively by JH and negatively by 20E, activates DILPs production in IPCs, they, in turn, stimulate AKH production in *CC*, and AKH ensures carbohydrate and lipid mobilization in the fat body.

## 7. The Effect of Short-Term Heat Stress on Feeding Behavior in *Drosophila melanogaster*

Changes in metabolic behavior can also be considered as a delayed response to heat stress. It has been shown that significant changes in feeding intensity as well as in lipid content in *D. melanogaster* females occur in 24 h after short-term heat exposure (38 °C, 60 min), resulting in a decrease in food consumption [48]. These data suggest that regulation of feeding behavior under stress may be controlled by means other than the IIS pathway, as the changes in the expression of the pathway’s genes and the increase in the carbohydrates level take place directly after short-term heat exposure, and the behavioral response manifests as a decrease in food consumption 24 h after the exposure, resulting, apparently, in a decrease in lipid content. This conclusion agrees with the idea that feeding regulation and glycaemia control are the result of complex interaction of metabolic, hormonal and neural signals that have not yet been fully elucidated [86]. 

The regulation of feeding behavior under heat stress seems to occur without the participation of the *dilp6* and *dfoxo* genes as their mutations do not prevent the decrease in the appetite after stress exposure in imagoes [48]. We assume that, in flies with a *dfoxo* mutation, the IIS pathway feedback loop (see Figure 1) is disrupted, and when in response to food intake the dFOXO signal becomes insufficient for modulating the expression of *dilp6* and *dInR*, the levels of which remain low, and for regulating other genes responsible for changes in feeding behavior as well. The existence of other mechanisms of feeding regulation, besides the IIS pathway, is evidenced by the fact that disruption of the next link of the “lesser” feedback loop, DILP6, causing similar changes in the level of *dilp6* and *dInR* expression, does not prevent the behavioral stress response of a decrease in feeding intensity [48]. 

DA signaling in the mushroom body, a major *Drosophila* memory center [87], can potentially be such a mechanism. So, it has been revealed that flies consume more food during long-term memory formation, and RNAi expression against DopR1 in the neurons of the mushroom body results in impairment of long-term memory [88]. In turn, a decrease in sucrose consumption and suppression of proboscis extension in *D. melanogaster* has been demonstrated under the activation of mushroom body output neurons [89].

A constitutive increase in the appetite of flies with *dilp6* and *dfoxo* mutations could be connected with the increase in DILPs production in IPCs caused by the abovementioned disruptions in the “lesser” feedback loop. This assumption is confirmed by the data on a higher DILP3 level in the mutant *dilp6*^41^ females with a decreased DILP6 function [21]. The ability of DILP6 to suppress DILPs production in IPCs is also demonstrated by the data on the decrease in both DILP2 secretion and *dilp2* and *dilp5* expression at the overexpression of *dilp6* in the adult fat body [9]. Thus, if DILP6, as a consequence of its mutation or a mutation of *dfoxo*, is incapable of inhibiting DILPs in IPCs, their levels increase, leading to higher fly appetite and the formation of the diabetic phenotype, including increased carbohydrate and lipid levels. The decrease in dInR expression in flies with a *dilp6* mutation is likely caused by constitutionally increased levels of DILPs synthesized in IPCs and plays a compensatory role. Another possible explanation of this phenomenon can be the ability of DILP6 to activate dInR, similarly to DILPs in IPCs, which leads to a decrease in the receptor’s expression at DILP6 dysfunction.

## 8. Conclusions and Future Perspectives

In recent years, many studies have been performed with the use of modern omics methods, such as transcriptomics, proteomics and metabolomics, undoubtedly providing a plethora of new data regarding the metabolic and other systems of the organism. However, the contribution of studies researching the endocrine regulation of metabolism with the use of classical methods—specific gene mutations and knockouts, pharmacological treatments with various hormones and tissue-specific expression regulation using the UAS-4 system—to elucidate the mechanisms of these systems’ functioning should not be underestimated. Here, we examine results from studies performed almost exclusively with the use of such methods: they reveal the complexity of the mechanism of the *D. melanogaster* imago neuroendocrine stress reaction, the interaction of its key elements such as biogenic amines (DA and OA), gonadotropins (JH and 20E), AKH and insulin-like peptides, and their role in regulating carbohydrate and lipid metabolism under heat stress conditions. We assume that further investigation in this field will help to unveil the connection of hormonal pathways involved in the stress reaction to elements of cellular stress response, including the c-Jun-N-terminal kinase (JNK) signaling pathway and the heat shock response pathway.

## Data Availability

Not applicable.

## Supplementary Materials

The following supporting information can be downloaded at: https://www.mdpi.com/article/10.3390/insects14050474/s1, Table S1: The time course of changes in the levels of hormones and biogenic amines and the activity of their metabolic enzymes in *Drosophila* under heat stress (38 °C) [22,90,91,92,93,94,95,96,97].
