# Supplementary material for: Mechanisms of Neuroendocrine Stress Response in Drosophila and Its Effect on Carbohydrate and Lipid Metabolism
Source: Insects. 2023 May 17;14(5):474. doi: 10.3390/insects14050474 (PMC10231120; doi:10.3390/insects14050474)
Supplement: Supplementary file 1 [file insects-14-00474-s001.zip › insects-2389912-supplementary.pdf]

Table S1. The time course of changes in the levels of hormones and biogenic amines and the activity of their metabolic enzymes in *Drosophila* under heat stress (38 °C).

Hormone/enzyme	Direction of changes	Time course, min	Reference
Dopamine	rise	15	Rauschenbach et al., 1993[1];<br>Hirashima et al., 2000[2]
Octopamine	rise	15	Hirashima et al., 2000[2]
20-hydroxyecdysone	rise	60	Hirashima et al., 2000[3]
Juvenile hormone	assumed rise		Gruntenko, Rauschenbach, 2018[4]
JH-esterase	drop	180	Gruntenko et al., 1999[5]
JH-epoxide hydrolase	drop	180	Gruntenko et al., 1999[5]
Ecdysone 20-monooxygenase	rise	60	Chentsova et al., 2007[6]
Thyrosine hydroxylase	drop<br>rise	30<br>60	Sukhanova et al., 1995[7]
Alkaline phosphatase	drop<br>rise	30<br>240	Sukhanova et al., 1995[7]
Tyrosine decarboxylase	drop	60	Sukhanova et al., 1997[8]
N-acetyl transferase	drop<br>rise	30<br>60	Rauschenbach et al., 1997[9]
DOPA decarboxylase	drop	30	Rauschenbach et al., 1997

[1] Rauschenbach, I.Yu., Serova, L.I., Timochina, I.S., Chentsova, N.A., Schumnaja, L.V. Analysis of differences in dopamine content between two lines of *Drosophila virilis* in response to heat stress. *J. Insect Physiol*. **1993**.*39*, 761–767.

[2] Hirashima, A., Sukhanova, M.Jh., Rauschenbach, I.Yu. Biogenic amines in *Drosophila virilis* under stress conditions. *Biosci. Biotechnol. Biochem*. **2000a**.*64*, 2625–2630.

[3] Hirashima, A., Rauschenbach, I.Yu., Sukhanova, M.Jh., . Ecdysteroids in stress responsive and nonresponsive *Drosophila virilis* lines under stress conditions. *Biosci. Biotechnol. Biochem.* **2000b** 64, 2657–2662.

[4] Gruntenko, N.E.; Rauschenbach, I.Yu. The role of insulin signalling in the endocrine stress response in *Drosophila melanogaster*: A mini-review. *Gen. Comp. Endocrinol*. **2018**, *258*, 134–139. https://doi.org/10.1016/j.ygcen.2017.05.019.

[5] Gruntenko, N.E., Khlebodarova, T.M., Sukhanova, M.Jh., Vasenkova, I.A., Kaidanov, L.Z., Rauschenbach, I.Yu.. Prolonged negative selection of *Drosophila melanogaster* for a character of adaptive significance disturbs stress reactivity. *Insect Biochem Mol Biol*. **1999**,*29*,445-452. https://doi.org/10.1016/s0965-1748(99)00021-1.

[6] Chentsova, N.A.; Gruntenko, N.E.; Rauschenbach, I.Yu. Ecdysone 20-monooxygenase activity in *Drosophila virilis* strains varying in ecdysteroid response to heat stress. *Russ. J. Genet*., **2007**, *43*, 829–830. https://doi.org/10.1134/S1022795407070174.

[7] Sukhanova, M.Z.; Grenback, L.G;.Gruntenko N.E.; Khlebodarova T.M.; Rauschenbach I.Y. Alkaline phosphatase in *Drosophila* under heat stress. *J. Insect Physiol*. **1995**, *42*, 161–165.

[8] Sukhanova, M.Z.; Grenback, L.G;.Gruntenko N.E.; Khlebodarova T.M.; Rauschenbach I.Y. Tyrosine decarboxylase and dopa decarboxylase in *Drosophila virilis* under heat stress. *Biochem. Genet.* **1997**, *35*, 91-103. https://doi.org/10.1023/a:1022209707655.

[9] Rauschenbach, I.Yu.; Sukhanova, M.Jh.; Shumnaya, L.V.; Gruntenko, N.E.; Grenback, L.G.; Khlebodarova, T.M.; Chentsova, N.A. Role of DOPA decarboxylase and N-acetyltransferase in regulation of dopamine content in *Drosophila virilis* under normal and stress conditions. *Insect Biochem. Molec. Biol*. **1997**, *27*,, 729–734. https://doi.org/10.1016/s0965-1748(97)00051-9.
